# The use of stereotactic body radiation therapy for local control of glomangiomatosis: a case report

**DOI:** 10.3389/fonc.2013.00026

**Published:** 2013-03-05

**Authors:** Zachary D. Horne, Sana D. Karam, Abdul Rashid, J. W. Snider, Allison Lax, Metin Ozdemirli, K. W. Harter

**Affiliations:** ^1^Department of Radiation Medicine, Georgetown University HospitalWashington, DC, USA; ^2^Department of Radiology, Georgetown University HospitalWashington, DC, USA; ^3^Department of Pathology, Georgetown University HospitalWashington, DC, USA

**Keywords:** SBRT, stereotactic body radiation therapy, stereotactic body radiotherapy, glomangiomatosis, glomus tumor

## Abstract

The vast majority of glomangiomas are small, benign neoplasms that can occur anywhere in the body but typically arise in the subcutaneous tissues of the extremities and are capable of causing extreme pain. Typically, these lesions are managed surgically with excellent rates of tumor control. On occasion, patients present with a variant of the glomangioma tumor consisting of numerous or recurrent nodules, a condition classified as glomangiomatosis. The authors present a case report of a young patient with multiply recurrent painful glomangiomas of the left foot, who was ultimately diagnosed with glomangiomatosis pedis. After multiple surgeries and surgical consultations, no surgery other than amputation was recommended. Therefore, the patient sought consultation with regard to stereotactic body radiation therapy (SBRT). In the absence of other options, and based on its effectiveness in treating glomus tumors of the head and neck which display similar natural history and histologic features, SBRT was offered. The patient underwent SBRT to the largest of his remaining tumors with excellent local control and significant reduction in pain at two and a half years follow-up.

## Introduction

Peripheral glomus tumors which most often present subungually in the digits of the upper extremities are primarily small, clinically benign neoplasms of the dermis and hypodermis (Folpe et al., 2001). They typically present with a classic triad of pain out of proportion to examination, temperature sensitivity, and point tenderness, though these symptoms are non-specific and not necessary for diagnosis. These lesions were better characterized by Masson in 1924 as arising from the glomus body, a neuromyoarterial apparatus responsible for thermoregulation (Masson, 1924). It has since been found that glomus tumors constitute less than 1% of all neoplasms of the soft tissue and usually occur in the extremities or sometimes in the jugular foramen (glomus jugulare) as singular nodules (Looi et al., 1999). Some patients may be afflicted with multiple glomus tumors, a condition known as glomangiomatosis. As a subset of glomus tumors, patients afflicted with glomangiomatosis tend to present with multiply recurrent peripheral tumors (Moor et al., 1999).

Herein, we describe the use of stereotactic body radiation therapy (SBRT) with anecdotal experience from the treatment of glomus jugulare in the therapy of a young patient with multiple and recurrent glomangiomas of the left foot.

## Case presentation

**Figure 1:**
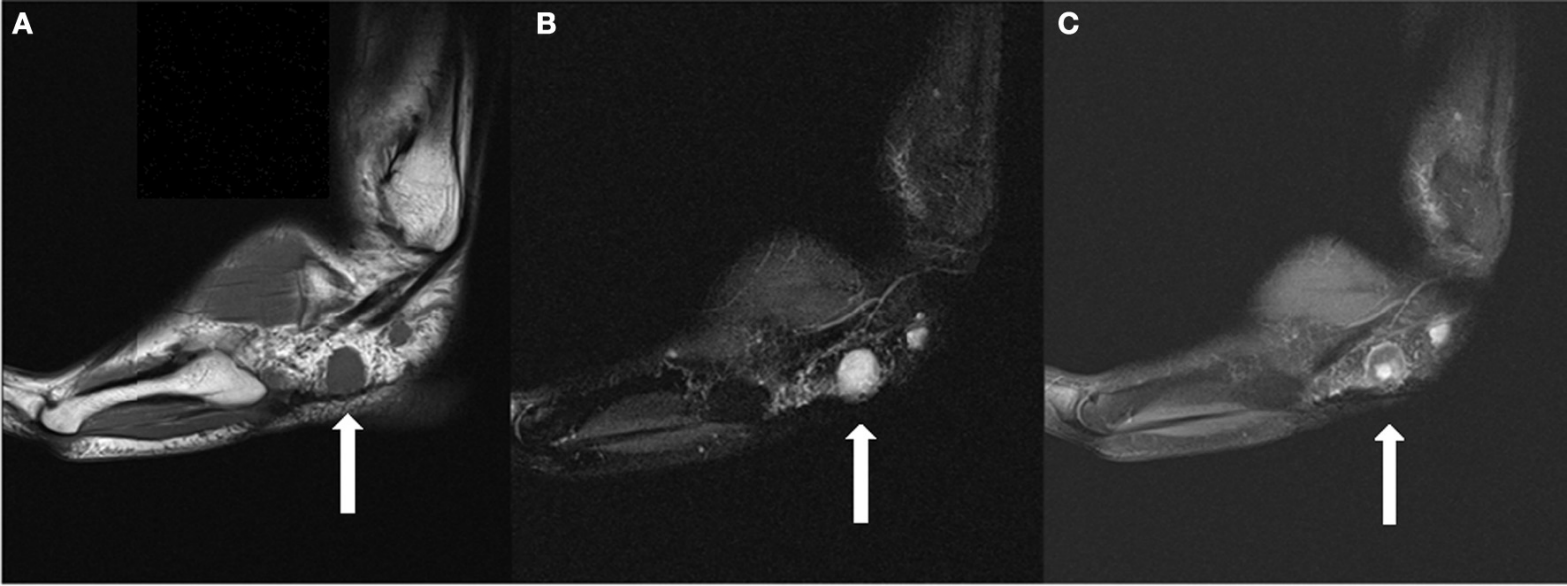
**(A)** Sagittal T1 weighted image demonstrates a 1.8 cm T1 isointense round mass in the lateral subcutaneous soft tissues, plantar to the peroneal tendons. **(B)** The lesion is hyperintense on T2 fat suppressed images. **(C)** The lesion demonstrates a small area of central contrast enhancement on T1 fat suppressed images (whereas it demonstrated homogenous enhancement on earlier studies). A smaller lesion is partially seen posterior to the dominant lesion.

A 28 year-old Caucasian male without otherwise notable past medical history presented with a 13 year history of pain secondary to multiple tumors of the left foot. He underwent his first surgical excision at the age of 17 after 2 years of progressively worsening discomfort. Pathologically determined to be a glomangioma, this lesion was removed from the posteriolateral ankle soft tissues. An additional lesion was resected 2 years later from the anterolateral heel. The patient underwent four additional surgeries at 2, 3, 4, and 6 years following initial presentation and was ultimately diagnosed with glomangiomatosis pedis. He had been referred for further surgery including possible amputation, which he declined. He initiated care with our institution ~6 years following the last surgery (12 years following initial presentation). His symptoms included increasing swelling and pain of his largest lesion over the lateral tarsal region of his left foot. The pain was described as consistent at 2 out of 10 in intensity, with regular exacerbations to 9/10 (Houde, 1982). Examination revealed an approximate 2 cm palpable tumor over the lateral aspect of the left foot at the level of the calcaneocuboid articulation. The patient experienced exquisite tenderness over the region of the sinus tarsi which correlated with a remaining surgical clip on radiographic examination. He had mild diminution in sensation near his surgical scars, but motor function remained intact. Computed tomography (CT)/Magnetic resonance imaging (MRI) revealed multiple soft tissue masses throughout the left mid and hindfoot, with the largest mass located in the lateral hindfoot and measuring 1.8 cm in greatest dimension, having grown from 1.37 cm 8 months prior and correlating to the location of his current symptoms (Figure 1).

**Figure 2:**
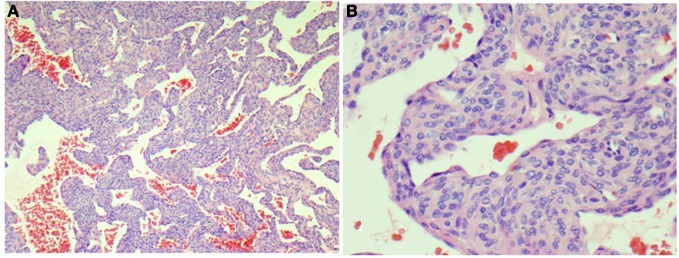
**Representative section from the left foot mass shows vascular tumor containing perivascular proliferation of small cuboidal epithelioid cells without atypia around open vascular spaces (HandE stains, A, 40×; B, 200×)**.

Histologic evaluation from the most recent surgical excision (6 years prior to presentation to our service) consisted of multiple samples, all consistent with glomangiomatosis (Figure 2). Microscopically, they were a collection of fibroconnective and fibromuscular soft tissues which appeared as focal limited vascular proliferation with increased perivascular cellularity.

As part of this patient's work-up, he was referred to three separate surgeons specializing in Podiatric, Plastic, and Orthopedic surgeries. The patient declined superficial peroneal nerve resection to remove the retained surgical clip. He was otherwise ruled inappropriate for further surgical intervention with the exception of amputation. With this, the patient was offered SBRT for local control of the largest and currently troublesome lesion. He was not offered therapy for the remainder of his lesions at the time of presentation for a number of reasons. As this modality had not been utilized for the treatment of a glomangioma of the foot, the results and consequences of SBRT to the hindfoot were relatively uncertain and our primary goal was to do no harm. We were concerned about the outcomes of offering additional treatment on the patient's multiply resected and resultantly fibrotic foot. Further, the remaining lesions were asymptomatic and the course of their progression was uncertain. If his remaining lesions were to progress and additional radiotherapy deemed necessary, there were concerns over cumulative dose to the bones of the foot and related pathological fractures as a late toxicity as well as increasing the level of fibrosis already present from prior surgical interventions.

He was counseled as to the relative dearth of literature regarding the use of this modality in the treatment of peripheral glomus tumors. The patient elected to proceed with SBRT for treatment of his symptomatic lesion.

## Irradiation technique

**Figure 3:**
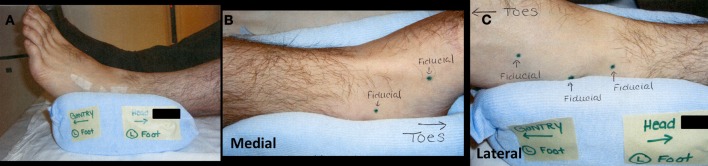
**(A)** Lateral view of immobilization device. Small strips of tape hold gold fiducials over placeholder tattoo markings. **(B)** Medial view of immobilization device with two placeholder tattoos. **(C)** Lateral view of immobilization device with three placeholder tattoos.

**Figure 4:**
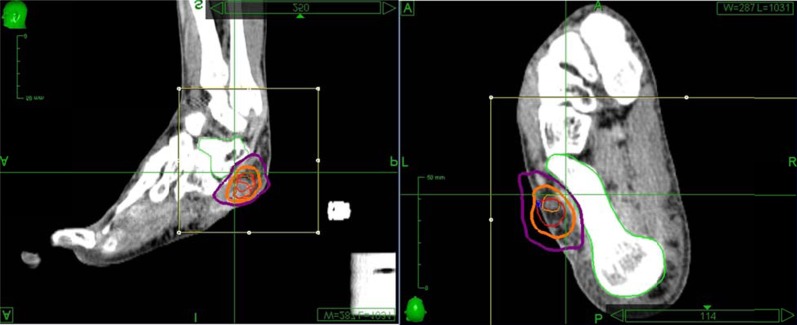
**The CyberKnife treatment plan utilizing-fused CT and MRI scans of the left foot.** The gross tumor volume was outlined on both scans and prescribed a dose of 3000 cGy. Thin orange is the CT-GTV, which was treated to a minimum of 3122 cGy and a maximum of 4139 cGy. Thin red represents the MRI-GTV, which was treated to a minimum of 3289 cGy and a maximum of 4225 cGy. Each GTV received 100% of the prescribed dose. The two GTVs were fused with an extension of 2 mm to create the CTV (thick orange) and a margin of 5 mm was added (thick purple) was added to create the PTV. The talus was outlined and kept to a maximum of 2260 cGy.

Treatment was delivered using the CyberKnife robotic SBRT system (Multiplan version 2.1.0, Accuray, Inc.) with 6-MV photons from a linear accelerator mounted on a fully articulated robotic arm. Due to the difficulty of implanting fiducials in the foot, five golden fiducials were affixed to the patient's foot at the time of CT simulation and tattoos were used to mark their positions (Figure 3). The fiducials were affixed to his foot over the tattoos at the time of obtaining the MRI for treatment planning and for each treatment session to monitor intrafraction tumor motion with orthogonal kV x-rays. Treatment was administered to the patient as an outpatient in five fractions over the course of 7 days. His lesion was treated on consecutive days, starting on a Wednesday and ending on a Tuesday, excluding the weekend. His foot was immobilized using a TriVac SecureVac Cushion (Bionix Development Corp.). Because of the tumor's location, the treatment was planned utilizing fused CT and MRI scans. The gross tumor volume (GTV) was outlined on each scan and on CT was 4.6 cm^3^ and on MRI was 3.2 cm^3^. The CT-GTV and MRI-GTV were fused with rigid registration and expanded by 5 mm to create the clinical tumor volume (CTV). The CTV was expanded by 2 mm to create the planning tumor volume (PTV) (Figure 4). The PTV was prescribed to 30 Gy in 6 Gy fractions to the 71% isodose line. Inverse planning was used to determine the dose to the target volume while minimizing the dose to normal tissue. Dose constraints to the talus were adapted from the 22 Gy threshold dose for ribs found in Benedict et al.'s (2010) guidelines for tissue constraints (Benedict et al., 2010). For the talus, a larger bone, a slightly higher threshold of 25 Gy was utilized. The minimum and maximum doses to the CT-GTV were 31.2 and 41.4 Gy, respectively. For the MRI-GTV, the minimum and maximum doses were 32.9 and 42.3 Gy, respectively. Each GTV received 100% of the prescribed dose. The talus was also outlined to quantitate the dose delivered, which was a maximum of 2260 cGy.

## Follow-up

**Figure 5:**
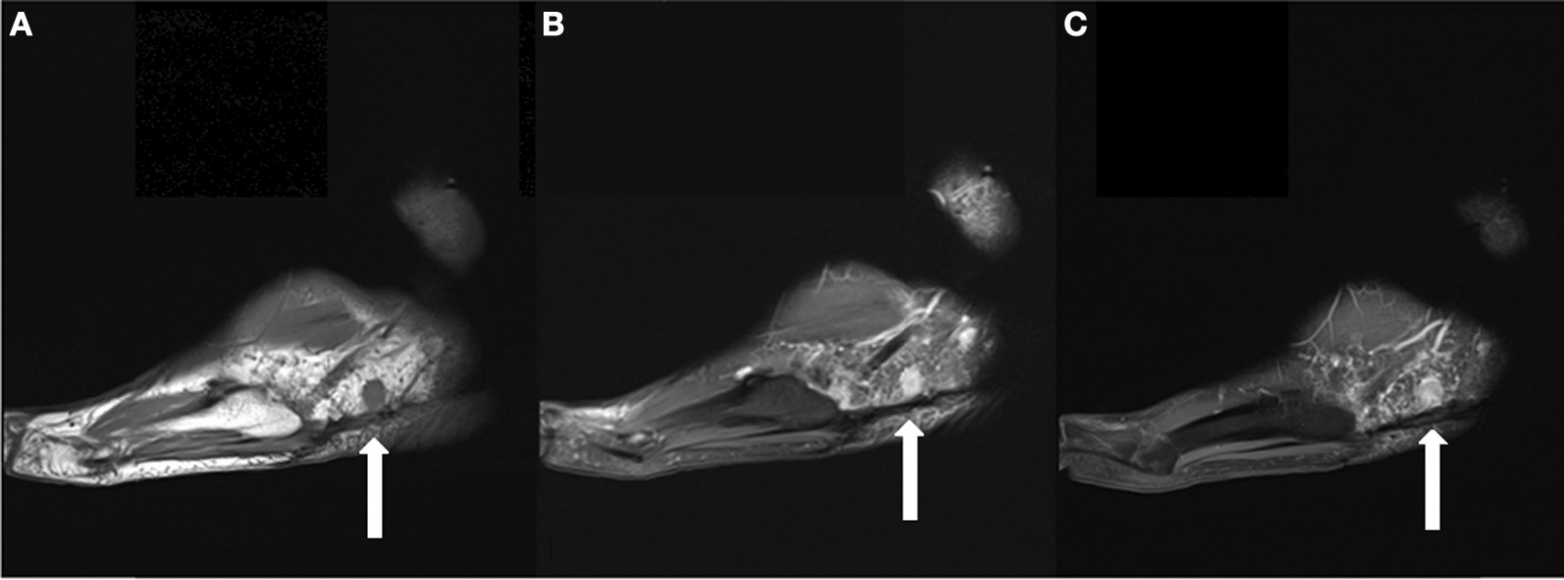
**(A)** Sagittal T1 weighted image obtained 6 months following the patient's SBRT treatment demonstrates interval decrease in the size of the dominant mass to 1.1 cm. **(B)** It remains hyperintense on T2FS images. **(C)** It also demonstrates homogeneous enhancement on T1 fat suppressed post contrast images.

**Figure 6:**
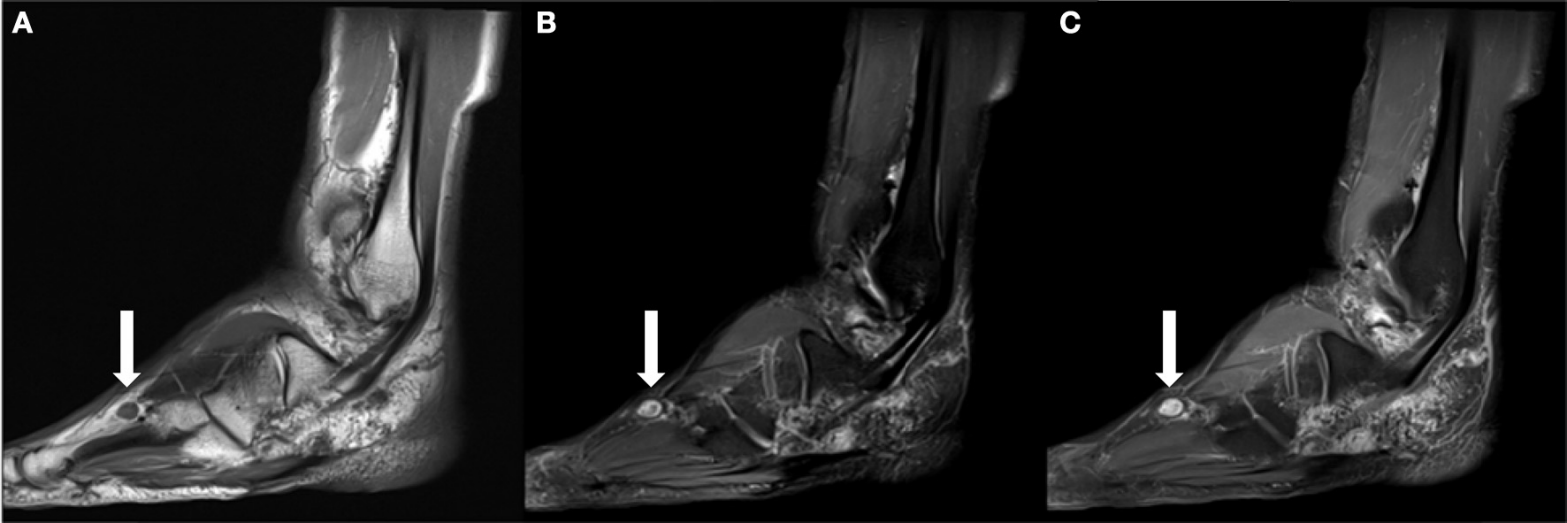
**(A)** Sagittal T1 weighted image obtained ~2 years following the patient's SBRT treatment demonstrates interval development of an enlarging T1 isointense nodule within the subcutaneous soft tissues dorsal to the fourth-metatarsal diaphysis. **(B)** It is hyperintense on T2 fat suppressed images. **(C)** Similar to previously identified masses, it demonstrates contrast enhancement on T1 fat suppressed post contrast imaging.

The patient tolerated therapy with no significant complications and only mild soreness over his instep which resolved shortly following completion of treatment. He followed up with our clinic at regular 6-month intervals, including MRI imaging of his left foot. His MRI at 6 months post-treatment showed no new lesions as well as a decrease in size of the target lesion to 1.1 cm in maximal dimension, a reduction of 0.7 cm (Figure 5). At 6 and 12 month follow-up, he noted a resolution of his baseline discomfort with significantly less severe exacerbations with a maximum of 4 out of 10 (9/10 prior to treatment). At 2.5 years of follow-up, the patient continued to endorse reduced pain at the site of the treated lesion. He did, however, complain of recent onset of new pains similar to those of the past on the dorsal surface of the left foot near the proximal diaphysis of the fourth-metatarsal. These pains were accompanied by the ability to palpate a nodule in the region of the pain. This is consistent with the most recent imaging, which showed an increase in size to 0.9 cm of an existing nodule seen on previous studies (Figure 6). Treatment of the new lesion was delayed at the patient's wishes.

## Discussion

In 2001, Folpe et al. proposed a classification scheme for glomus tumors with the following categories: malignant glomus tumor, glomus tumor of uncertain malignant potential, symplastic glomus tumor, and glomangiomatosis (Folpe et al., 2001). Under this new system, glomus tumors were differentiated from glomangiomas by their histological similarity to angiomas (Folpe et al., 2001). In contrast to solitary glomus tumors, glomangiomas more closely resemble venous malformations than the glomus body (Schopp et al., 2009). Additionally, the natural history of the glomangioma is also distinct from the glomus tumor. Again in contrast to those with solitary glomus tumors, patients with glomangiomatosis tend to present at a younger age and with peripheral tumors that are rarely subungual (Moor et al., 1999). There has also been evidence to suggest that there is a familial component to glomangiomatosis, with patients inheriting the trait in an autosomal dominant fashion (Shotton et al., 1987).

For most glomus tumors, embolization followed by surgical excision remains the first-line intervention (Schopp et al., 2009). Stereotactic radiosurgery and SBRT have also been used to treat solitary, residual, and recurrent glomus jugulare tumors with good results (Lim et al., 2007; Wegner et al., 2010; Guss et al., 2011; Ivan et al., 2011; Sheehan et al., 2012). An extensive literature search revealed only one documentation of external beam radiation therapy being utilized to treat multiple peripheral glomus tumors (Nishimoto et al., 1990). To our knowledge, SBRT has not been reported in the management of glomus tumors or their variants in the extremities, making our use of the technique for the treatment of glomangiomatosis unique.

The incidence of glomangiomatosis is estimated to be 2 in 1,000,000 and one-third of these cases will present before the patients are 20 years old (Goodman and Abele, 1971; Duggan et al., 2008). Glomangiomas can present in any location, such as the extremities, trachea, lungs, nasal cavity, stomach, kidneys, and others (Al-Ahmadie et al., 2007; Alempijevic et al., 2008; McKenna and Kerr, 2008; Miyamoto and Wada, 2009; Parker et al., 2010; Rao et al., 2010; Santambrogio et al., 2011). Though glomangiomas have the potential to present anywhere in the body, they most often present in the extremities (Anakwenze et al., 2008; Glazebrook et al., 2010). Because this condition is rare but often debilitating due to the pain of the tumors, surgical treatment is usually necessary for problematic lesions. In cases of glomangiomatosis, this can lead to disfigurement from multiple procedures on a small area and may ultimately lead to amputation of a digit or limb. In an effort to avoid this outcome, radiation therapy has been investigated as an alternative treatment modality.

Focused radiation therapy has been shown to at least control, if not resolve symptoms resulting from glomus tumors, especially the glomus jugulare (Nishimoto et al., 1990; Lim et al., 2007; Wegner et al., 2010; Guss et al., 2011; Ivan et al., 2011; Sheehan et al., 2012). In choosing an appropriate treatment dose, two factors were considered: the success of previously reported treatment schemes and the possibility of late toxicity from treatment. Two series from the University of Pittsburgh and Stanford University utilizing SBRT for the treatment of glomus jugulare tumors reported excellent outcomes using 16–25 Gy in 1–5 fx and 14–27 Gy in 2 or 3 fx (Lim et al., 2007; Wegner et al., 2010). In two series from Princess Margaret Hospital related to the treatment of lower extremity soft tissue sarcoma with conventionally fractionated external beam radiation, pathological fracture rates were as high as 10% in patients treated to greater than 60 Gy and risk of fracture was elevated when the mean dose to the bone was greater than 37 Gy or maximum point dose was greater than 59 Gy (Holt et al., 2005; Dickie et al., 2009). Further, the Bendict et al.'s 2010 guidelines were taken into account, ensuring maximum point doses to bony structures were less than 30 Gy (Benedict et al., 2010).

Given that the patient was being treated to a relatively small area adjacent to the talus and the possibility of the patient requiring future SBRT to other progressing tumors, we chose to treat only the dominant lesion to a dose of 30 Gy. Following treatment to the dominant lesion, the patient was able to return to running regularly without pain or discomfort. This is not only a testament to the success of the treatment, but also to the fact that the other lesions, while present, were asymptomatic. In the event that the patient does require further SBRT to the left foot in the future, he will certainly be at an increased risk of pathological fracture with increased cumulative dose to the bones of the hindfoot. The local control was excellent as not only did the progression of his most problematic lesion halt as would have been expected based on the glomus jugulare literature, but it reduced in size by 0.7 cm without any side effects other than a short-lived tenderness in his foot. Patients with multiply recurrent glomangiomas of the extremity, SBRT may provide a limb sparing alternative with excellent local control and minimal toxicity. Due to the small sample size and retrospective nature of the report, this technique warrants further investigation to delineate the role of SBRT in the treatment of glomangiomas.

### Conflict of interest statement

The authors declare that the research was conducted in the absence of any commercial or financial relationships that could be construed as a potential conflict of interest.
